# Efficient algorithms for the discovery of gapped factors

**DOI:** 10.1186/1748-7188-6-5

**Published:** 2011-03-23

**Authors:** Alberto Apostolico, Cinzia Pizzi, Esko Ukkonen

**Affiliations:** 1 Dipartimento di Ingegneria dell'Informazione, Università degli Studi di Padova, Padova, Italy; 2 College of Computing, Georgia Tech, Atlanta, USA; 3 Department of Computer Science, University of Helsinki, Helsinki, Finland; 4 Helsinki Institute for Information Technology, Helsinki, Finland

## Abstract

**Background:**

The discovery of surprisingly frequent patterns is of paramount interest in bioinformatics and computational biology. Among the patterns considered, those consisting of pairs of solid words that co-occur within a prescribed maximum distance -or *gapped factors*- emerge in a variety of contexts of DNA and protein sequence analysis. A few algorithms and tools have been developed in connection with specific formulations of the problem, however, none can handle comprehensively each of the multiple ways in which the distance between the two terms in a pair may be defined.

**Results:**

This paper presents efficient algorithms and tools for the extraction of all pairs of words up to an arbitrarily large length that co-occur surprisingly often in close proximity within a sequence. Whereas the number of such pairs in a sequence of *n *characters can be Θ(*n*^4^), it is shown that an exhaustive discovery process can be carried out in *O*(*n*^2^) or *O*(*n*^3^), depending on the way distance is measured. This is made possible by a prudent combination of properties of pattern maximality and monotonicity of scores, which lead to reduce the number of word pairs to be weighed explicitly, while still producing also the scores attained by any of the pairs not explicitly considered. We applied our approach to the discovery of spaced dyads in DNA sequences.

**Conclusions:**

Experiments on biological datasets prove that the method is effective and much faster than exhaustive enumeration of candidate patterns. Software is available freely by academic users via the web interface at http://bcb.dei.unipd.it:8080/dyweb.

## Background

The computation of statistical indexes containing subword frequency counts, expectations, and scores thereof, arises routinely in the analysis of biological sequences. This problem is usually manageable when the word length is limited to some fixed, small value but rapidly escalates in complexity when applied on a genomic scale, perhaps without any length bound. In principle, a sequence of *n *characters may contain Θ(*n*^2^) distinct substrings, whence an exhaustive statistical index would be by one order larger than its subject. In previous work by [1], the size of such exhaustive tables has been shown to reduce to *O*(*n*) by a prudent combination of properties related to pattern maximality and monotonicity of scores. In informal terms, maximal substrings in a sequence may be obtained by partitioning all sub-strings into equivalence classes, in such a way that the strings in each class share precisely the same set of starting positions in that sequence. Thus, every word in a class must be a prefix of some *maximal *word *w*, that together with the list of occurrences represents the entire class. A classical result bounds the number of such representatives by *O*(*n*). In addition, it has been shown that the *z-scores *or departure from expected occurrence count of the elements in each class are monotonically increasing. This allows one to score only the representative in each class, since any other word in that class is a prefix of, and not more surprising than, the representative. Similar *conservative *approaches have been already applied successfully to patterns affected by indeterminacies of various kinds [2,3].

In this paper, we consider the problem of exhaustive counting and discovery of pairs of subwords that co-occur more frequently than expected within a specified distance in a sequence. In the literature, these patterns are also refered to as *gapped factors*.

In [4], and [5], indexes are proposed to solve the problem of *searching *a text for an assigned gapped-factors query. The problem of *discovering *such signals is intrinsically more difficult than searching, since no specific query is provided as input, and all candidate patterns must be weighed by some score of statistical significance.

Within the bioinformatics literature, algorithms from [6-9], have been proposed for the closely related problem of discovering *composite motifs*. These are indeed combinations of two or more *approximate *signals that may not have an exact occurrence in the input sequences. Hence those algorithms have been designed to compute a slightly different statistic than ours. In [10] the exhaustive enumeration of gapped factors has been proposed for the detection of dyadic transcription factors.

In contexts other than bioinformatics, a generalization of gapped factors was addressed in [11] under the name of *word association pattern*, which refers to a tuple of *k *components matching the input string within distance *d*. The algorithm takes time *O*(*d*^*k*^*n*^*k*+1 ^log *n*), which reduces to our problem by setting *k *= 2, yielding time *O*(*d*^2^*n*^3 ^log *n*), or *O*(*n*^3 ^log *n*) when *d *is a constant. A related algorithm was also proposed in [12], running in time *O*(*n*^3^) but requiring *O*(*n*^2^) scans of the input string, for the case of gapped factors.

We show that *O*(*n*^2^) time and space suffices to build and represent this kind of statistical index using a compact approach for both *head-to-head *and *up to d head-to-tail *distances. An additional linear factor is to be charged when dealing with *up to d tail-to-head *distance or if *d *is a parameter. Furthermore, the computation of *z*-scores can be included in our constructions within the same complexity bounds.

Our presentation is organized as follows. We first recapture previous results on optimal count for gapped factors within head-to-head distance, and then present algorithms dealing with other distance measures between factors. We also show how to incorporate in the computation *z-scores*, extending the framework to a discovery process based on over-representation, and the space savings induced by their monotonicity within gapped factors equivalence classes.

Finally, we test our approach on the problem of discovering spaced-dyads and compare the efficiency and efficacy of our *compact *approach with respect to the exhaustive enumeration of [10].

## Methods

### Problem statement

**Figure 1:**
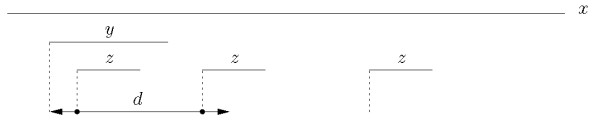
**Basic head-to-head index**. Assuming there are no other occurrences of *y *and *z *in *x*, for a given distance *d *it is *I_HH _*(*y*, *z*) = 2.

**Figure 2:**
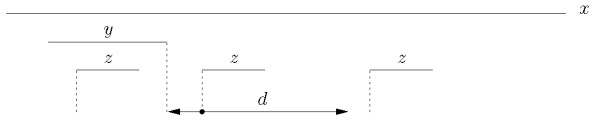
**Basic tail-to-head index**. Assuming there are no other occurrences of *y *and *z *in *x*, for a given distance *d *it is *I_TH _*(*y*, *z*) = 1.

**Figure 3:**
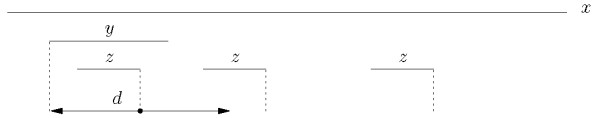
**Basic head-to-tail index**. Assuming there are no other occurrences of *y *and *z *in *x*, for a given distance *d *it is *I_HT _*(*y*, *z*) = 1.

Let *x *be a string over some alphabet Σ, where |*x*| = *n*, and let *d *be some fixed non-negative integer. Given two strings *y *and *z *that occur in *x*, the triplet (*y, z*, *d*) defines a *gapped factor *where *y *is the first component, *z *is the second component, and it is asked that they co-occur at a distance less than or equal to *d*. When *d *is fixed we just refer to the pair of factors (*y*, *z*). For any pair (*y*, *z*) of strings in *x*, the co-occurrence count is the number of times an occurrence of *z *follows an occurrence of *y *within a distance *d*. The variety of ways in which the distance between components may be measured leads to several variants for the problem, as exemplified in Figure 1, 2, and 3.

**Definition 1**. The *basic head-to-head index I_HH _*(*y*, *z*) relative to *x *is the number of times that *z *has an occurrence in *x *within a distance *d *from an occurrence of *y *to its left.

**Definition 2**. The *basic tail-to-head index I_TH _*(*y*, *z*) relative to *x *is the number of times that *z *has an occurrence in *x *within a distance *d *≥ 0 from the last symbol of an occurrence of *y *to its left.

**Definition 3**. The *basic head-to-tail index I_HT _*(*y*, *z*) relative to *x *is the number of times that *z *has an occurrence in *x *which ends within a distance *d *from a corresponding occurrence of *y *to its left.

We can also require that no interleaving occurrence of one or the other factor occurs between the considered occurrences of *y *and *z*. We then talk about the *relaxed tandem index Î*(*y*, *z*), and the *tandem index I*(*y*, *z*), for which the head-to-head distance are defined as follows.

**Definition 4**. The *relaxed tandem index Î*(*y*, *z*) relative to *x *is the number of times that *z *has an occurrence in *x *within head-to-head distance *d *from a corresponding, closest occurrence of *y *to its left.

**Definition 5**. The *tandem index I*(*y*, *z*) relative to *x *is the number of times that *z *has a closest occurrence in *x *within head-to-head distance *d *from a corresponding, closest occurrence of *y *to its left.

**Figure 4:**
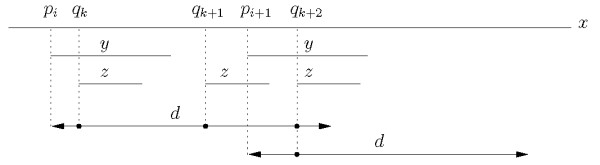
**Interleaving occurrences**. Illustrating interleaving occurrences.

We illustrate the definitions of tandems for head-to-head distance in Figure 4.

Let *p*_*i *_and *p*_*i*+1 _be the starting positions of the only two occurrences of *y *in an input sequence, and let *q*_*k*_, *q*_*k*+1 _and *q*_*k*+2 _be the starting positions of the only three occurrences of *z*. Let *d *be the maximum distance allowed for co-occurrences, as sketched in Figure 4. The basic head-to-head index for (*y*, *z*) is *I*_*HH *_(*y*, *z*) = 4: three co-occurrences corresponding to the occurrence of *y *at *p*_*i *_and one corresponding to the occurrence of *y *at *p*_*i*+1_. Hence, the set of co-occurrences is {(*p*_*i*_, *q*_*k*_), (*p*_*i*_, *q*_*k*+1_), (*p*_*i*_, *q*_*k*+2_), (*p*_*i*+1_, *q*_*k*+2_)}.

To obtain the relaxed tandem index *Î*(*y*, *z*) only the occurrences of *z *that fall within distance *d *from an occurrence of *y*, but before the next occurrence of *y*, are counted. In the case of Figure 4 this produces a contribution of two pairs for the first occurrence of *y*, and one for the second. In fact, the last occurrence of *z *is now counted only once, in association with its closest occurrence of *y *at *p*_*i*+1_.

Finally, to obtain the tandem index *I*(*y*, *z*), one more adjustment is needed, since now only one of the two occurrences of *z *that pair up with *p_i _*is to be counted, namely, the closest one. This yields the final value of *I*(*y*, *z*) = 2, from the two co-occurrences {(*p_i_*, *q_k_*) and (*p*_*i*+1_, *q*_*k *+2_)}.

### Algorithms

Here we present algorithms to count and discover the various kinds of gapped factors defined in the previous section. The index for computing *I_HH _*with and without interleaving occurrences was first introduced in [3]. We add to this the description of notable variants and the discovery frameworks.

As it was mentioned earlier, since there may be Θ(*n*^2^) distinct substrings in *x*, then the number of possible gapped factors, either interleaving or not is Θ(*n*^4^), and such is the time necessary for their exhaustive enumeration. Our algorithms exploit some properties of the suffix tree data structure to reduce this complexity. A suffix tree *T_x _*of an input string *x *defined over an alphabet Σ is a compact trie of all the suffixes of *x*$, where $ is a special symbol that does not belong to Σ. Suffix trees can be constructed in time linear in the length of the input string. We refer to [13-15] for detailed constructions. In our discussion, we use the letters *v*, *x*, *y*, and *z *to denote strings, and the letters *α *and *β *for nodes of the tree. The word ending precisely at vertex *α *of *T_x _*is denoted by *w*(*α*), and *α *is called the *proper locus *of *w*(*α*). The *locus *of a substring *v *of *x *is the unique vertex *α *of *T_x _*such that *v *is a prefix of *w*(*α*) and *w*(*Father*(*α*)) is a proper prefix of *v*.

**Figure 5:**
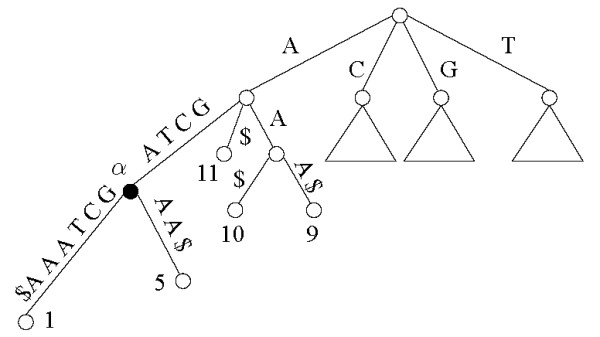
**Suffix tree**. Partial Suffix Tree for the string AGCTAGCTAAA. The words AG, AGC, AGCT, and AGCTA share the same locus *α*, hence they occur starting at the same positions {1,5} in the text.

Suffix trees enjoy several interesting properties [16,17]. In particular, since in any such tree there are exactly *n *leaves (one for each suffix) and each internal node is a branching node, then the total number of nodes is linear. By the structure of the tree, the occurrences of a word *v *start precisely at the suffixes that are found in the subtree rooted at the locus of *v*. Thus, any word *v *ending in the middle of an arc will have the same starting positions, and consequently the same number of occurrences, as the word *w*(*α*) with *α *the locus of *v *(see Figure 5).

#### Counting Gapped Factors: Head-to-Head distance

**Figure 6:**
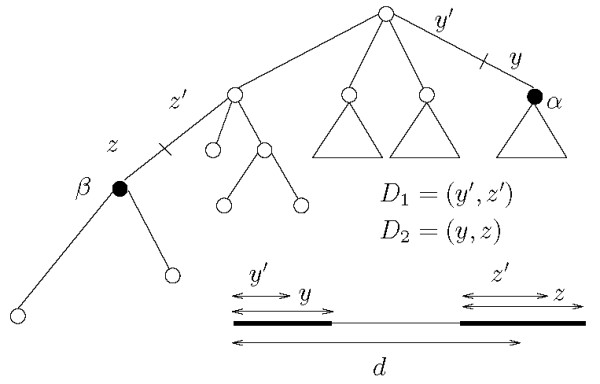
**Maximal classes**. Some maximal classes in the suffix tree *T_x_*.

The *O*(*n*) words ending at the branching nodes and leaves of the suffix tree represent a compendium of all the substrings of the input string *x*, and each node or leaf represents an equivalence class, in the sense that strings with the same locus share the same set of starting positions. Clearly, it is enough to consider as factors only pairs of class representatives, where the representative of a class is the longest string in that class. For any pair of words (*y'*, *z'*) not explicitly considered, there exists another pair (*y*, *z*), formed by representatives, hence such that *y' *is a prefix of *y*, and *z' *is a prefix of *z*, and the two pairs have the same number of co-occurrences as shown in Figure 6. Hence, for a given query (*y'*, *z'*) we can report as result the value of *I_HH _*(*y*, *z*).

The occurrences for gapped factors that, within a given distance *d*, have a fixed string *y *as their first component are computed in three steps as follows:

a) Consider the starting positions *p*_1_, *p*_2_,..., *p_k _*of all occurrences of *y *in *x*, where *y *= *w*(*α*) and *α *is a node in *T_x_*,

b) Let *L *be the mapping leading from each sequence position to the corresponding leaf node, and give a weight to each leaf, initially set to zero. For each *p_i _*mark the positions *p_i _*+ 1, *p_i _*+2,... *p_i _*+*d*, and add one to the weight of the corresponding leaf nodes {*L*(*p_i _*+1), *L*(*p_i _*+ 2),..., *L*(*p_i _*+ *d*)};

c) Traversing the tree bottom-up, annotate each internal node *β *with the sum of the weights of the children nodes.

Upon completion, the generic node *β *of the tree holds the value of *I_HH_*(*y, z*), where *z *= *w*(*β*). The annotation of the tree for a fixed *y *takes *O*(*n*) time if *d *is fixed. Since this has to be repeated for each one of the *O*(*n*) choices of the first component *y*, then the overall execution time is *O*(*n*^2^). Indeed, with a properly tuned construction of the index, the time can be linear in the output size [18].

#### Counting Gapped Factors: Tail-to-Head distance

**Figure 7:**
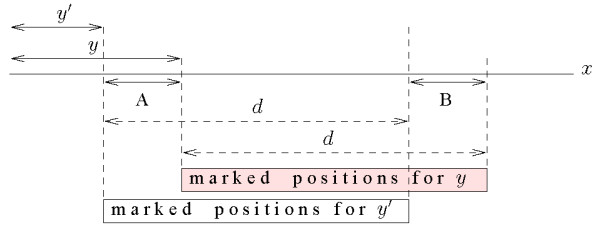
**Boundaries for tail-to-head distance**. The tail-to-head distance cannot be computed by marking up to *d *positions from the tail of the maximal string corresponding to *y'*: the positions falling within segment *A *would not be counted, while those in segment *B *would be counted while they should not.

When we consider tail-to-head distance, we cannot directly apply the previous method and just add a shift of length |*y*| to the marking phase. In fact, the prefix *y' *of *y*, that has the same locus *α *in the tree as *y *will be at a tail-to head distance from a certain *z *larger than the distance between *y *and *z*. Thus, when we mark the positions *p_i _*+ |*y*| + *j - *1, 1 ≤ *j *≤ *d*, as in Figure 7 some positions (those falling in segment *A*) that should be marked for *y' *will not be marked, and some other (those falling in segment *B*) that should not be marked for *y' *will be actually marked. Note that even though *A *and *B *have clearly the same length the strings that they intercept are generally different.

This problem is overcome by building, in time and space *O*(*n*^3^), the index  between maximal strings *for all *possible non negative *exact *distances *h*. The index is built as follows:

a) Consider the starting positions *p*_1_, *p*_2_,..., *p_k _*of all occurrences of *y *in *x*, where *y *= w(*α*) and *α *is a node in *T_x_*;

b) Let *L *be the mapping leading from each sequence position to the corresponding leaf node, and give a weight to each leaf, initially set to zero. For each *p_i _*add one to the weight of the leaf node *L*(*p_i _*+ |*y*| + *h*);

c) Traversing the tree bottom-up, annotate each internal node *β *with the sum of the values of the children.

At the end of this computation the generic node *β *of the tree holds the value of  where *z *= *w*(*β*). The annotation of the tree for a fixed *y *and *h *takes *O*(*n*) time. This process needs to be repeated for every distance 0 ≤ *h < n *and for every choice of the first component *y*, taking overall *O*(*n*^3^) time and space.

**Figure 8:**
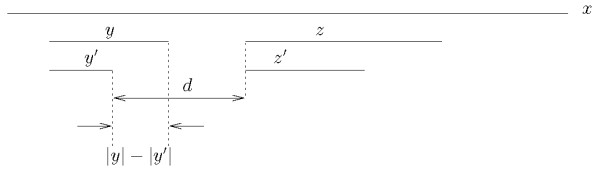
**Computing the tail-to-head index (case a)**. Computing the number of co-occurrence tail-to-head of *y' *and *z' *at distance *d *from those of *y *and *z *when |*y'*| + *d *>|*y*|.

**Figure 9:**
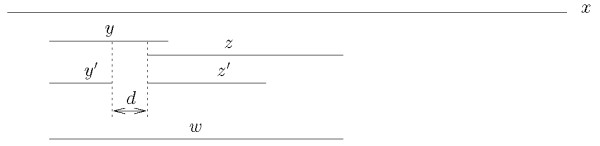
**Computing the tail-to-head index (case b)**. Computing the number of co-occurrence tail-to-head of *y' *and *z' *at distance *d *from those of *y *and *z *when |*y'*| + *d *≤|*y*|.

This set of trees can then be used to compute the tail-to head index between any two strings, not necessarily maximal, occurring in the sequence. Let *y' and z' *be two strings that have respectively *y *and *z *as the strings corresponding to their loci. For a query *I_TH_*(*y', z'*) within distance *d *one needs to compute , 0 *≤ h ≤ d*. For each *h *we need to distinguish two cases:

1. |*y'*| + *h *> |*y*|: the occurrences of the corresponding maximal strings *y *and *z *do not overlap, hence  (see Figure 8).

2. |*y'*| + *h *≤ |*y*|: the occurrences of the corresponding maximal strings y and *z *overlap. Let *w *be the string given by the concatenation with overlap of *y *and *z' *(see Figure 9): *y*_1 _... *y*_|*y'*|+*h *_· *z'*. We can get the number of co-occurrences directly by searching the occurrences of *w *in the suffix tree.

The time required by these two operation is *O*(|*y*| + |*z'*|). The tail-to-head index is finally given by .

#### Counting Gapped Factors: Head-to-Tail distance

This case consists of gapped factors in which the distance is measured from the beginning of the first component to the end of the second.

The case of head-to-tail distance is a very special case. In fact here we are interested in counting the number of strings that fall completely within a bounded distance *d *from the beginning of the first component.

Let us build the suffix tree for the input sequence. For a first component *y *= *w*(*α*), occurring at *n_y _*positions  in *x *we consider the set of substrings {*x*[*p_i _*+ 1, *p_i _*+ *d *- 1], *i *= 1.. *n_y_*}. We then build a generalized suffix tree for , where each $*_i _*is an end-marker ($*_i _*≠ $*_j _*if *i *≠ *j*) needed to correctly compute the index. The total length of these strings, and so the order of the number of nodes in the tree, is (*d *+ 1) × *n_y_*.

For each string *z *= *w*(*β*), where *β *is a node of this tree, the count of the number of leaves of the subtree rooted at *β *will give the number of co-occurrences between *y *and *z *at head-to-tail distance *d*. In this count all the substrings of *y *are also counted. If we want to avoid to include this obvious type of co-occurrence, we can mark the leaves coming from the positions within the occurrence of *y *while building the tree, and avoid counting them.

Obviously, if a string *z *= *w*(*β*) is totally contained within distance *d *from an occurrence of *y*, so will be any of its prefixes *z' *with locus *β*. On the other hand, every prefix *y' *of *y *with locus *β *such that *y *= *w*(*α*) will share with *y *the same set of starting positions. In the light of these considerations, a general query *I_HT _*(*y'*, *z'*) will be answered with the indexed value *I_HT _*(*y*, *z*).

In terms of time complexity, we have that for each string of the type *y *= *w*(*α*),*α *∈ *T_x _*with *n_y _*occurrences we perform *O*(*d *× *n_y_*) operations. This must be repeated for every node *α *∈ *T_x_*. The number of occurrences of each node are given by the sum of the occurrences of its children. In the worst case (think of the tree for *a^n^*$) their sum is *O*(*n*^2^). So the total complexity is *O*(*d *× *n*^2^). Since *d *is constant, we have *O*(*n*^2^).

#### Avoiding interleaving occurrences

The computation of the tandem indexes of *z *and *y *prescribes that two kinds of intermediate occurrences be avoided, as described next.

#### Computing the Relaxed Tandem Index

To compute the relaxed tandem index, each oc-currence of *z *must be referred to its closest occurrence of *y *to the left. The algorithm to compute the relaxed tandem index is similar to the basic algorithm, where steps (a) and (c) are unchanged, while in step (b) only the positions *p_i _*+1, *p_i _*+2,... *p_i _*+ *d'*, where *d' *= min{d, *p*_*i*+1 _- *p_i _*- 1} are marked. Time and space complexities of the basic algorithm stay unchanged.

#### Computing the Tandem Index

**Figure 10:**
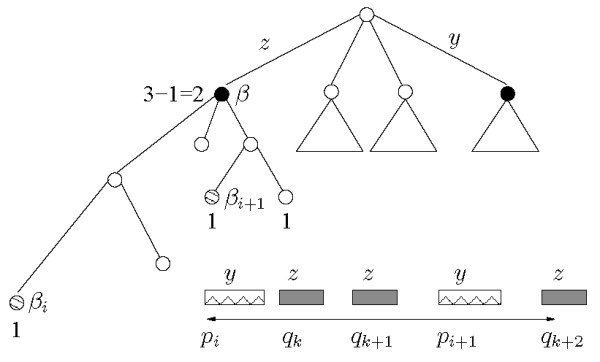
**Avoiding interleaving occurrences**. If *L*(*q*_k_) = *β*_*i *_and *L*(*q*_*k*+1_) = *β*_*i*+1_, a unit decrement must be taken from the index of the node *β *= *LCA*(*β*_*i*_, *β*_*i*+1_).

To compute the tandem index we first apply steps (a) and (b) for the construction of the relaxed tandem index. Furthermore, for each occurrence *p_i _*of *y *we create the list of nodes . For every pair of consecutive nodes (*β*_*i*_, *β*_*i*+1_) in the list, we invoke the LCA algorithm of [19] in order to identify their least common ancestor node *β*, and we subtract 1 to its weight. This adjustment is needed because in the computation of the relaxed index the longest common prefix *z *between nodes *β_i _*and *β*_*i*+1_, rep-resented by node *β*, has been weighed twice, once from the path that leads to the leaf *β_i_*, the other from the path that leads to *β*_*i*+1_. This is true also for any node in the path from the root to *β*. So step (c) is needed to propagate both the weights and the adjustments. The weight of an internal node is then given by the sum of the weights of its children with the possible subtraction of the value weighted during the LCA calls. An example is shown in Figure 10 where the suffixes starting at *q*_*k *_and *q*_*k*+1 _share the common prefix *z *= *w*(*β*).

The strategy to avoid interleaving occurrences was first presented in [3]. Independently, [20] presented a similar approach to count gapped factors in a set of sequences. In their work the LCA algorithm is used to take into account just one instance per sequence.

#### Discovering Over-represented Gapped Factors

Let *y *and *z *be the first and second factors in a pair, respectively, *d *the distance between them, and assume that *y *and *z *do not overlap. We take the expected frequency of *D *= (*y*, *z*) to be the product of the expected frequencies of the individual terms *y *and *z*, formally: *F_e_*(*D*) = *f*(*y*)*f*(*z*), and consider two possible settings, depending on whether *f*(*y*) and *f*(*z*) are computed on the input sequence *x *or on a given external (super)sequence. In both cases it may be argued along the lines of [1] that it suffices to weigh the pairs corresponding to the nearest branching nodes, that correspond to the representatives of equivalence classes the score of which cannot be smaller than that of any other pair from the same classes.

Consider two substrings *y' *and *z' *of *x *with no proper locus in *T_x_*. Let *α *and *β *be their respective loci, and let *y *= *w*(*α*) and *z *= *w*(*β*) be the corresponding strings (cf. Figure 6).

Clearly, *y' *and *y *share the same set of starting positions, and the same holds for *z' *and *z*. In fact, this property holds for all pairs of strings having respectively *α *and *β *as proper locus. Hence these strings can be said to belong to an equivalence class, let it be denoted by *C_yz_*, in the sense that the number of times that they co-occur coincides with the number of times that *y *and *z *co-occur. I.e., *F_c_*(*D'*) = *F_c_*(*D*), where *D' *= (*y'*, *z'*) and *F_c_*(·) is the frequency count.

Moreover, under i.i.d. hypothesis we have for the expected frequency *F_e_*:

In other words, for all gapped factors *D' *= (*y'*, *z'*) in *C_yz_*, the probability of *D' *= (*y'*, *z'*) is not smaller than that of *D *= (*y*, *z*).

Consider now the following scores comparing expected and counted number of co-occurrences:

1. 

2. 

3. 

4. 

It follows from our discussion that since, moving downward along an arc, *F_c _*remains constant while *F_e _*is non-increasing, then the value of any such score is non-increasing, whence it is enough to compute it only for pairs of strings having a proper locus.

We conclude that it suffices to score only the representative for each class, since the pair *D*(*y*, *z*) of strings with a proper locus will attain the highest score among members of *C_yz_*.

## Results and Discussion

As an example application we demonstrate the performance of our method in connection with the discovery of co-occurrences representing transcription factor binding sites. It is known that these sites have a variable structure, although they are all characterized by a high degree of similarity among their oc-currences. Many pattern discovery algorithms (see, e.g., [21] and references therein) assume that the errors are randomly distributed in the pattern. However, this is not necessarily the case. The variability among binding sites might be concentrated at their centers or on their sides. For example, the factor Gal4p, that belongs to the zinc finger class, binds to a pair of conserved regions, separated by a fixed length segment of DNA of variable content. This kind of binding sites are also called a *spaced dyad*.

In [10], the problem of dyad discovery is solved through an algorithm based on exhaustive enumeration of pairs of components. A significance score is computed with respect to a given background model, and used to rate the dyads. The output is given by the pairs with the highest scores. This approach was proved to be effective in the prediction of such spaced dyads, however, the exhaustive enumeration of dyads, the computation of the background and the scanning of the sequence for each candidate makes the computation imposing and not scalable with the size of the components.

To test our approach on the dyad discovery problem we implemented a simplified version of the head-to-head and tail-to-head algorithms. The software, called *DyVerb *was developed in Java and it is available via a web interface at http://bcb.dei.unipd.it:8080/dyweb/.

In a direct emulation of the RSAT dyad-analysis tool by [10], we applied *DyVerb *to the computation of the co-occurrences between all the TST leaves at tail-to-head distance varying from 0 to 16, and the expected frequency of the dyad is given by the product of the counted frequencies of the components. The length of the components was set to 3, and we searched both strands. We then performed two runs of experiments to verify both the efficacy in the discovery of the dyad signals using several scores, and the execution time performance. These experiments are described in the following subsections.

### Efficacy of discovery on real datasets

We considered as benchmark the dataset from [10], which consists in 8 gene families that are regulated by zinc-bicluster transcription factors. In Table 1 we report the results in the extraction of spaced dyads based on the scores *z*_2 _and *z*_3 _and also the significance score defined in [10], with monadic back-ground frequencies taken from the input sequences. For completeness, we report here the definition of the significance score:

**Table 1:** Efficacy on dyad discovery

Efficacy of Discovery		
	**rank**	**motif**

GAL4	1/1/1	**CGG**RnnRCYnYnCn**CCG**

CAT8	15/3/3	**CGG**nnnnnn**GGA**

HAP1	7/1/6	C**GG**nnnTAn**CGG**(nnnTA)

LEU3	1/1/1	R**CCG**Gnn**CCG**GY

LYS	1/3/3	WWW**TCC**RW (T|**C**)**GG**AWWW

PDR	1/2/2	TYT**CCGCGG **ARY

PPR1	1/1/1	WY**CGG**nnWWYK**CCG**AW

PUT3	1/1/1	Y**CGG**nAnGCnAnnn**CCG**A

UGA3	3/1/1	AAA(A|**G**)**CC**GC (G|**C**)**GG**CGGSAWT

UME6	2/1/1	T**AGCCGC**CGA

Discovery of dyad motifs by DyVerb for length 3 and distance up to 16. For each family, the second column displays the rank of the score values in the format *z*_2 _/*z*_3 _/*significance *of the corresponding motif. The latter appears in superposition to the actual site, highlighted in bold.

where *P *(*D*, ≥ *n*) is the probability of observing at least *n *occurrences of the dyad *D *in the input set, and *N_p _*is the number of unique spaced dyads in which the components have length 3 and distance varying from 0 to 16.

The table reports, for each set of sequences, the rank of the discovered motif which is closest to the real one, which is represented on the right with the gapped factor discovered by *DyVerb *highlighted in bold. In most of these tests, *DyVerb *ranked the gapped factors corresponding to known motifs at the top.

Note that several motifs can score the same value. The table lists such motifs following no particular order. This is irrelevant for most of the experiments, in which indeed the real motif commands the first position. For more subtle motifs such as CAT8, the score *z*_2 _ranks the motif as 15th, although the real position in the table is 24th. The value of *z*_3 _for the same motif is the 3rd largest, but it appears at the 6th position in the table. Similarly, for the significance score, the motif is 3rd in rank, but shown at the 5th position. We also found that for this motif all scores we computed resulted in similar values. Another subtle motif is HAP1. The best score here appears to be *z*_3_, however, for this score only two values were found, namely, 0.002 and 0.001. Nonetheless, only 18 motifs scored 0.002, and among those the gapped factor GGAnnnnnCGG that can be mapped easily to the real motif.

### Efficiency of discovery on real datasets

Additional experiments were performed for the purpose of assessing scalability. For this, we implemented exhaustive enumeration using the same programming language (Java) and the same machine (a Pentium4 running at 2.26 GHz with 1 GB of memory) as used for *DyVerb*. We noted the times required to compute different scores, specifically *z*_2_, *z*_3_, and *Signif*, the significance score. Table 2 shows the results of these experiments. It can be seen that for large datasets, such as UME6, the time required by *DyVerb *is significantly shorter than that required by the exhaustive approach.

**Table 2:** Efficiency on dyad discovery

Efficiency of Discovery				
	**DyVerb**			**Exhaustive**

	***z*_2_**	***z*_3_**	**Signif**	**Signif**

GAL4(5)	3.383	4.046	3.339	17.96

CAT8(4)	3.138	4.011	3.301	17.445

HAP1(9)	4.667	5.958	4.308	30.757

LEU3(5)	3.222	3.987	3.281	19.139

LYS(6)	3.593	4.566	3.796	21.226

PDR(7)	3.981	5.169	4.222	26.446

PPR1(3)	2.316	2.819	2.242	11.976

PUT3(2)	1.93	2.287	1.755	8.42

UGA3(3)	2.53	2.891	2.318	11.361

UME6(23)	8.338	10.08	11.773	75.09

Running times in seconds of DyVerb for *z*_2_, *z*_3 _and *Signif *scores, and of the exhaustive approach for *Signif*. The number of sequences in each family is reported in parenthesis.

## Conclusions

In this paper we presented algorithms based a on a conservative approach to the construction of statistical indexes for the discovery of over-represented co-occurrences. The advantage over exhaustive enumeration is in the substantial reduction of the space of candidates, which unlike heuristic approaches, does not pose the risk of missing the optimal ones. The web tool *DyVerb *was developed to discover dyadic motifs.

Experiments on real datasets showed that the simple probabilistic model used by *DyVerb *is capable of discovering known signals, in a simple and fast way. Future developments will address on one hand the optimization of the data structures in use, in order to deal with larger sequences, on the other, the implementation of more advanced probabilistic models such as those previously developed in [1] in connection with single patterns.

## Competing interests

The authors declare that they have no competing interests.

## Authors' contributions

AA, CP and EU jointly contributed to the conception, design, analysis of the algorithms, and jointly contributed to the writing and editing of the manuscript. Implementation and testing were done by CP. All authors read and approved the final manuscript.
